# Off label use of a 7Fr endo-biliary forceps system for endovascular inferior vena cava biopsy

**DOI:** 10.1186/s42155-025-00548-9

**Published:** 2025-04-21

**Authors:** Stavros Grigoriadis, Stavros Spiliopoulos, Athanasios Korogiannos, Konstantinos Palialexis, Dimitrios Filippiadis, Nikolaos Kelekis

**Affiliations:** 1 2nd Department of Radiology, Interventional Radiology Unit, National and Kapodistrian University of Athens, Attikon” University General Hospital, 1st Rimini St, 12461 Chaidari, Athens, GR Greece; 2 Interventional Radiology-Oncology Clinic, Athens Medical Center, Marousi, Athens, Greece; 3 Mediteraneo” General Hospital, Glyfada, Greece

**Keywords:** Endovascular biopsy, Inferior vena cava, Biliary forceps, Renal cell carcinoma

## Abstract

Endovascular inferior vena cava (IVC) mass biopsy emerges as a minimally invasive promising technique for the acquisition of tissue samples for histological analysis, crucial for determining the malignant or benign nature of the lesion and guiding subsequent treatment. This report details the first successful off label use of a low-profile 5.2Fr biliary forceps system, via a 7Fr sheath, for endovascular IVC biopsy in a 61-year-old male patient with a history of left nephrectomy due to Gravitz tumour presenting with IVC thrombosis at 5-years follow up, suspicious for recurrence.

## Introduction

Intravascular filling-defect lesions pose a significant diagnostic challenge in patients with suspected or confirmed malignancy. Very often, a percutaneous transabdominal or trans-lumbar approach is technically infeasible or carries a high risk of complications. Endovascular inferior vena cava (IVC) mass biopsy emerges as a promising technique [1]. This minimally invasive procedure enables the acquisition of tissue samples for histological analysis, crucial for determining the malignant or benign nature of the lesion and guiding subsequent treatment. This report details the first successful off label use of a low-profile 5.2Fr biliary forceps system, via a 7Fr sheath, for endovascular IVC biopsy.

## Case presentation

**Fig. 1:**
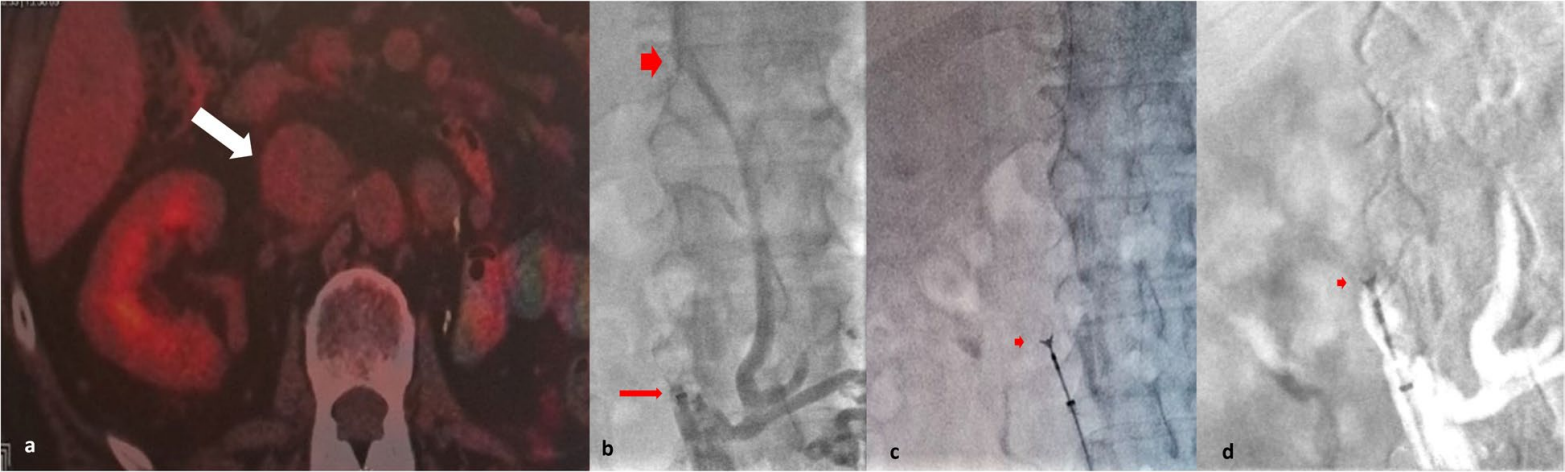
**a** 18F-FDG PET/CT image indicating the low 18F-FDG uptake of the IVC thrombosis (arrow). **b** Angiographic image indicating the sheath of the kit (arrow) within the proximal segment of the thrombosed IVC at the level of L1-L2 vertebra. Note that the thrombosis extends up to the level of T12vertebra (arrowhead). **c** Fluoroscopic and (**d**) roadmap images demonstrating the opened forceps (arrow) at the mid segment of the lesion, just before sampling

A 61-year-old male patient with a history of left nephrectomy performed on September 2019, due to Gravitz tumour (clear cell renal cell carcinoma according to the post-operative surgical biopsy). He presented with IVC thrombosis involving the right renal vein of the single right kidney, detected during regular CT imaging at 5-years follow up. As discussed at the oncologic multidisciplinary team meeting, the clinical question was to differentiate between IVC thrombosis and cancerous IVC invasion due to possible recurrence, as to promptly proceed with systemic therapy. The 18F-fluorodeoxyglucose positron emission tomography/computed tomography (18F-FDG PET/CT) scanning was inconclusive, due to its low 18F-FDG uptake (Fig. 1a). After carefully reviewing the radiological imaging, a percutaneous CT-guided biopsy was deemed of high risk for bowel perforation and extremely technically demanding, if not impossible, due to inability to safely access the IVC at the level of the renal artery, via a trans-abdominal or trans-lumbar route. The bleeding risk, although moderate, was also considered. Therefore, an endoluminal IVC forceps biopsy was decided due to the safest access and practically zero bleeding risk. Laboratory tests were within normal range (Ht: 39.5%, Hb: 13.1 g/dL; WBC: 8.7 × 10^3^/μl, PLTs: 183 × 10^3^/μl; INR 1.13). The patient was already treated with low molecular weight heparin, which was discontinued the day before and commenced the day after the biopsy. Access was obtained from the right common femoral vein, using local anesthesia (lidocaine 2%, 10mls) and a 21G/4Fr micro-puncture system (Merit Medical, USA). A standard J 0.035’’ guide wire was advanced within the IVC at the proximal segment of the occlusion and the 7Fr x 30 cm sheath of the set was placed at the proximal segment of the IVC lesion. The guide wire was retrieved and subsequently the 5.2Fr x 60 cm transluminal biliary biopsy forceps (BBFS; Cook Medical, Bloomington, IN, USA) designed to capture a 2.23 mm3 sample, was advanced within the proximal and middle part of the occlusion. Under continuous fluoroscopic guidance and using the “cross and push” technique [2], eleven (11) tissue samples were collected from various sites within the thrombotic filling defect and submitted for histopathological examination. Additionally, aspiration of material from an angled 5Fr angiographic catheter was performed (Vertebral, Cordis, USA) and the obtained thrombotic material was sent for cytological analysis. Haemostasis was achieved through manual compression, followed by 3 h of bed rest. The patient was subsequently discharged. Tissue samples, ranging in size from 0.05 to 0.15 cm, were analysed by the pathology laboratory. Histopathological examination, including immunohistochemistry, revealed findings consistent with primary clear cell renal cell carcinoma with no lymphocyte penetration. Immunohistochemical staining demonstrated positivity for Vimentin, Keratin 8/18, CD10, and P504S, while Keratin 7 was negative. Thin prep cytological analysis did not detect malignancy. The patient initiated treatment immediately following the diagnosis so as to turn the “cold” tumour to “hot” tumour with dual immunotherapy agents (nivolumab and ipilimumab).

## Discussion

This is the first case of endoluminal IVC biopsy using the specific biliary forceps set. The advantage of the endovascular approach is the ease and safety of access, as well as significantly reduced bleeding risk, since the IVC is not directly punctured, while even in the event of tumoral bleeding, it is likely to be contained within the IVC, similar to the rationale behind trans-jugular liver biopsies. The specific 5.2Fr forceps system is compatible with 7Fr sheaths and is designed to capture a 2.23 mm^3^ tissue specimen. However, specimens obtained in this case were up to15 mm in size (volume in mm^3^ was not available from lab analysis) and were sufficient for immunohistology diagnosis. Although the literature contains only three recent publications describing five successful cases of endovascular IVC biopsy using forceps, other systems typically employ larger-diameter forceps. Specifically, Lai et al. reported a successful case using an 8Fr endocardial biopsy forceps system [3] and Mohammed et. al recently reported another successful case using the Micro-Tech single-use 8.5 mm biopsy forceps, (Nanjing, China) [4]. Pomoni et al., reported three cases in which diagnosis was obtained using another 7Fr system (Bi-Pal, Cordis Corporation, Miami, FL, USA) [5]. Furthermore, one unsuccessful sampling case has been reported using an endomyocardial straight 6.4F biopsy forceps [6]. Based on previous reports, 10-French introducer sheaths should be preferred to allow continuous simultaneous aspiration during the biopsy manoeuvres to prevent distal embolization [5]. In this case, sheath upsizing to allow simultaneous aspiration was not deemed necessary as the sample was obtained from the proximal and middle site of thrombosis and distal embolization through the more distal occlusion was therefore not possible. Notably, additional thrombus aspiration used for cytologic analysis was not diagnostic and therefore tissue sampling should always be considered in such cases to increase the possibility of successful diagnosis. Using the endovascular approach, the operator was comfortable with obtaining multiple (*n* = 7) samples, minimizing the possibility of non-diagnostic results as the risk of bleeding was practically non-existent. Finally, although the endovascular technique has been described since 2018, its adoption in clinical practice remains limited. Among the possible causes could be the fact that forceps such as these herein described, are only recently available in IR departments. The need for IR physicians to request unfamiliar equipment from other departments likely impedes widespread use. The authors suggest to incorporate the endovascular technique in the IR training curriculum and determine clear indications such as “lesions inaccessible percutaneously” as to increase the penetration of the method in routine clinical practice. Conclusively, the specific low-profile 5.2Fr biliary forceps biopsy set was successfully and uneventfully used for endoluminal IVC biopsy. Prospective studies are required to support its endovascular use and to determine the optimal biopsy set size.

## Data Availability

All relevant material is available from the corresponding author on reasonable request.
